# The Corona crisis and climate protection—keeping long-term goals in mind: Cleverly directing economic aid and exploiting synergy potentials for urgently needed investments

**DOI:** 10.1007/s00550-020-00494-1

**Published:** 2020-06-03

**Authors:** Manfred Fischedick, Uwe Schneidewind

**Affiliations:** 1 Wuppertal Institute for Climate, Environment and Energy gGmbH, Döppersberg 19, 42103 Wuppertal, Germany; 2 Bergische Universität Wuppertal, Wuppertal, Germany

## The Corona crisis and climate protection—the three phases of crisis management for a long-term concept

Scientific know-how is currently being mobilised worldwide at full speed to better understand the medical mechanisms of COVID-19 propagation and to develop a suitable vaccine. The technological options for working from home, digital coordination and virtual learning are currently experiencing a completely new push. At the same time, the economy is moving towards a crisis situation that threatens to dwarf the 2008/2009 financial crisis. Companies and freelancers are facing a dramatic challenge—in maintaining supply chains and production, but especially in dealing with collapsing demand. At the same time, a mix of policy measures for short-term economic stabilization is emerging, which again exceeds the scope of emergency measures taken after the financial crisis.

Politically and institutionally, the state intervenes at the national and regional levels in ways never practiced before: entry bans, bans on events and assemblies, business closures and even curfews. The demands on the political leadership especially in open and democratic societies are immense. This is due to the fact that the Corona crisis in particular requires an enormous amount of solidarity. 80% of the population, who have only minor health problems to fear from the virus, show solidarity with 20% in particularly vulnerable population groups. The fabric of values in our societies is facing a major test—especially if emergency measures are to last for a long time.

**Fig. 1:**
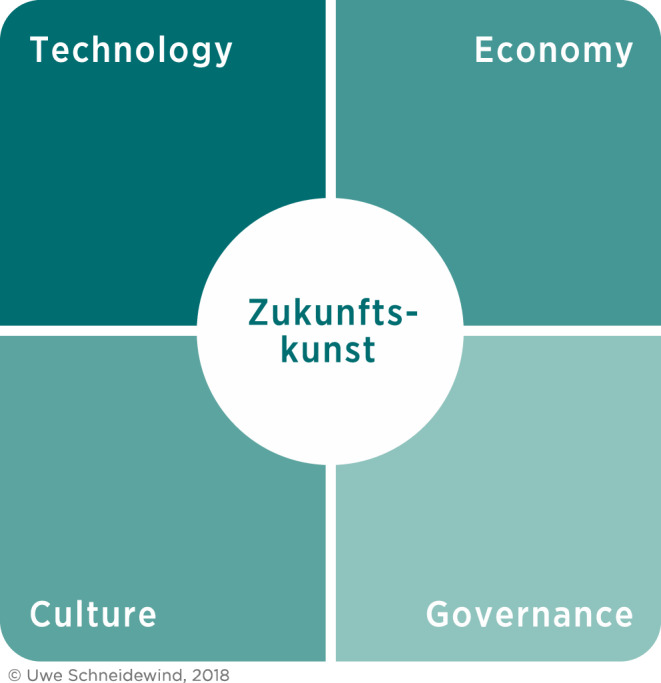
The four dimensions of “Zukunftskunst” as a guideline for shaping the future in the Corona crisis. (Schneidewind and Wuppertal Institute 2018) (The English translation of “Die Große Transformation” will be published at the end of 2020 by the Wuppertal Institut)

The examples outlined above show that the Corona crisis challenges the ability to shape the future in a special way. The Wuppertal Institute’s guiding framework for a “Zukunftskunst”, a future literacy, associates technological with economic, political and cultural changes and thus also provides orientation in the current crisis. Presumably there has never been more demand for “Zukunftskunst” in politics, economy and society than there will be in the coming weeks and months (Fig. 1).

*One thing must not be forgotten: The Corona crisis is hitting the world at a time when a large number of enormous challenges have to be solved.* This applies to the correct handling of the growing number of refugees and the elimination of social inequalities as well as, from an environmental policy perspective, to coping with the consequences of climate change and mitigating further changes in the climate. In addition, there are questions such as how to manage the transition to the digital age and how to close material cycles by means of increasingly circular forms of the economy—examples of long-term trends in which everyone (currently) finds themselves. In all areas, massive investments and a proactive design of the associated, sometimes massive, structural changes are required.

**Fig. 2:**
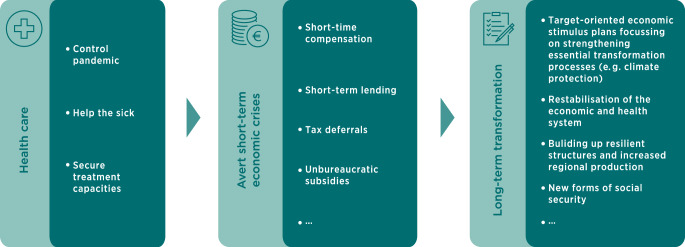
Three-phase model of dealing with the Corona crisis

Against this background, it is important not to lose sight of the long-term challenges for the future when dealing with the Corona crisis. Three phases are likely to characterise the management of the Corona crisis (see Fig. 2):

1. In the coming weeks, the focus will undoubtedly be first and *foremost on health care*. The aim is to contain a global Corona pandemic and avert hundreds of thousands of deaths. That is why health protection measures are currently so important.
2. At the same time, the massive *economic consequences have* to be *absorbed in the short term *with appropriate instruments. These include the measures that have now been adopted by many countries, such as the short-time working allowance, easier access to credit, tax deferrals and state guarantees, as well as direct payments to affected groups, such as the assumption of rental costs, compensation for loss of production costs or income. This must ensure that companies remain capable of acting and can contribute to the economic recovery after the crisis.
3. The third phase is *long-term crisis management* and refers to the aspects that are related to the economic and structural need for action resulting from the effects of the Corona crisis. These range from economic stimulus packages to revive the economy to fundamental structural adjustments, for example in the design of global value chains or a crisis-proof development of social security systems. It is important to think about this third phase at a very early stage, while keeping a firm eye on other global transformation challenges such as climate change. Early planning and a holistic perspective can avoid some of the failures in dealing with the consequences of the financial and economic crisis of 2008/2009.

This paper is intended to provide the first building blocks for this third phase. It was published in German on 20 March 2020 by the Wuppertal Institute. Comments and contributions to the discussion are welcome.

## Cleverly directing economic aid and exploiting synergy potentials for urgently needed future investments

In the third phase, it is particularly important to skillfully direct the necessary economic aid and to systematically exploit synergy potentials for investments that are necessary anyway. *If economic stimulus packages are launched as a follow-up to or accompanying the Corona crisis, it is important that these are developed with a direct focus on the future and that the funds spent are not distributed without explicit targeting. In other words: clear criteria are needed to which the measures can be aligned.* Whereas, in accordance with the phase concept (cf. Fig. 2), the short-term aid required has a rapid effect and must therefore be pragmatically oriented, the longer-term aid measures offer a unique opportunity not only to trigger economic stimuli but also to be able to set clear accents in the sense of a sustainable and robust design of the economy and society.

## Status of climate protection efforts and remaining challenges—case study Germany

To avoid an abstract discussion the situation in Germany is selected here as illustrative example (case study), but most of the mechanisms apply to all industrialised countries that have been struggling to meet their climate targets.

In climate protection, there is still a clear gap between objectives and reality. This is true regardless of the recent significant reduction in CO_2_ emissions or the CO_2_ equivalent emissions in the years 2018 and 2019 considered here. CO_2_ equivalent emissions include not only CO_2_ emissions but also emissions of other greenhouse gases—such as methane and nitrous oxide—which, for the most part, have a much greater impact on the climate than CO_2_ as a greenhouse gas. In Germany, emissions fell by 6.3% last year alone. In absolute terms, emissions fell by 54 mio. t CO_2_e between 2018 and 2019 and were still at 804 mio. t at the end of the year. The majority of 51 mio. t of CO_2_e can be traced back to the energy industry sector, again due to a combination of three effects:

- a significant increase in the price of CO_2_ certificates—partly as a result of adjustments to the rules in the European Emissions Trading System, i.e. the market stability reserve,
- a sharp drop in natural gas prices
- and an intensifying discussion on how to shape the phase-out of coal-fired power generation (including the transition of the first coal-fired power plants to so-called security readiness).

Overall, this led to a displacement of coal-fired power plants in the electricity mix in favour of natural gas power plants and renewable energies. In 2019, we dealt with an extremely good wind and sun year, so that the contribution of renewable energies to electricity generation increased, although in contrast to previous years (also due to politically uncertain framing) only a smaller increase in capacity was recorded.

Overall, German greenhouse gas emissions fell by 35.7% between 1990 and 2019. If the potential effects of the Corona crisis are taken into account, it does not appear unrealistic that a significant reduction in emissions in 2020 would lead to a reduction of 40% or more compared to 1990 and thus to formally achieve the national climate protection target for this year. For the potential effects to be expected from the Corona crisis are already clearly perceptible after just a few days.

*Which energy-economic and climate-political effects of the Corona crisis are to be expected or are already obvious?* First and foremost, industrial production is declining drastically, as evidenced by entire plant closures in the automotive industry. This reduces industrial energy requirements which can be seen very clearly in the electricity demand, which in turn leads to reduced emissions in the provision of energy/electricity. Initial experience from Italy and France shows that, as a result of the very drastic, but nevertheless necessary, measures taken at the beginning of March, the demand for electricity fell by 10% (France) and 20% (Italy) compared with the reference values from previous years. Incidentally, this is also in line with experience gained during the financial market and economic crisis in 2009. In Germany, the effects have been less severe so far, but here too there is already a tendency for demand to fall, with corresponding effects on the electricity price, which fell by around a third within a few days on the Leipzig Power Exchange (EEX) in mid-March (the figures apply to short-term futures contracts). In addition, mobility-related emissions have also been reduced, for example as a result of the increased transition to the home office, even though clear figures are still missing. *Is climate protection on the right track and can everyone sit back and relax? The clear answer is no, for several reasons:*

- The Corona effect will hopefully remain a one-off effect, and there will probably even be a catch-up development with increased emissions if it is possible to quickly limit the spread of the virus and gradually return to normality.
- Even the sharp declines in greenhouse gas emissions in 2018 and 2019 cannot be sustained and repeated as often as desired. These are partly due to one-off effects or would require great political courage to repeat on a similar scale. This is because a decline on the scale of 2019 has not yet occurred in Germany outside the economic crisis of 2009. These one-off effects include a five- to sixfold increase in CO_2_ certificate prices in both years, winter months with extremely mild temperatures, and a pull-forward effect on the closure of coal-fired power plants.
- And thirdly, there is a strong imbalance between the sectors that contribute to mitigation and others for which there is a clear need to catch up. The latter applies to the building sector but even more so to the transport sector. While emissions in the building sector have increased by 4.4% from 2018 to 2019, the increase in the transport sector was only 0.7%. It should not be overlooked though that the transport sector is the only sector where CO_2_ emissions have not fallen but have actually risen since 1990.

Therefore, there is no reason to lose sight of the climate tasks, neither with regard to the climate balance for 2020, nor for the target for 2030, by which time emissions must be reduced by 55% compared to 1990 according to the current legal basis. This target may also increase slightly if, in the course of the discussion in the coming months, the European Commission concludes that it will raise its target for 2030 (previously 40% compared with 1990) to 50 or even 55%. It is hardly conceivable that this can be done without adjusting the national German target. To this extent, *the future will require more rather than fewer measures, a high degree of continuity and extensive investment*. There is therefore a strong case for an economic stimulus package by the EU, the German federal government and the German federal states in the aftermath of the Corona crisis (i.e. in the third phase shown in Fig. 2) to address this issue precisely and attempt to utilise such synergy effects. Boosting the economy and stimulating employment can be achieved at various levels, such as investment in the renovation and insulation of houses, the expansion of renewable energies or the accelerated conversion to electric vehicles, to name just a few examples. Just how successful a clever investment programme can be in conjunction with continuous political and social support is demonstrated by the success story of the expansion of renewable energies since the Renewable Energy Sources Act (EEG) was passed 20 years ago. Starting from a share of electricity generation of just over 5% in 2000, this had already risen to over 42% by 2019.

However, the measures must go far beyond this and state investments, especially in the industrial sector, must be used to make the various sectors (especially energy-intensive industry) ready for the future. This includes among other things

- the conversion of steel production to hydrogen-based (green) production processes
- the entry into a hydrogen economy as a whole,
- the successive but consistent closing of material cycles within the framework of a circular economy,
- the electrification of transport (e.g. of overhead line structures for freight transport along motorways)
- the complete change to an entirely renewable power/energy supply^1^,
- and the consistent use of digitisation as an enabler for the central transformation arenas of our time (such as the energy, mobility, industrial, urban, consumption and food transition).

There is no question that this will bring about further massive structural changes in our economic cycles (including those induced by the Corona crisis). *But when, if not now—in times that are extraordinary for the economy anyway—should be a very good opportunity to accelerate and proactively accompany the transformation processes that are necessary anyway?*

## What is at stake now

*It is time, otherwise there is a great danger that one crisis will be replaced by another massive global crisis—the climate crisis.* We can already be sure that the climate will continue to change and that we will have to get used to higher temperatures in Germany as well. On the basis of new forecasting models based on statistical estimates, the German Weather Service expects that by 2029 it could become 1.5 to 2 ℃ warmer on average in Germany than the average of the last three decades. But we still hold the key to action. It should be used for the benefit of the climate and ultimately also for the benefit of German industry, which can take on a pioneering role, to position itself for the future through climate protection and climate adaptation measures and thus also to firmly establish itself in the climate protection markets of the future.

## Further long-term transformation challenges

In view of the long-term transformation challenges, it is also important to seize the opportunity to learn further lessons from the Corona crisis. This applies:

1. to the reflection of consumption and behavioral patterns,
2. the reduction of vulnerabilities through globally networked value chains in central production areas and
3. the crisis-proof provision of products and services of general interest and basic services, such as goods for the health system.

Already in the first weeks of the crisis new forms of digital work and digital learning are gaining considerable importance in practice—we are also learning how they can be integrated into everyday life in the long term.

The crisis also shows under which conditions people are prepared to make considerable adjustments and show solidarity. All of this will improve our understanding of societies in comprehensive transformation processes and will also be helpful for the climate debate.

However, it is also clear that technical and economic systems must become more robust. This applies, for example, to the resilience of infrastructures (e.g. energy infrastructures), which form the backbone of our society. The Corona crisis shows how vulnerable many of our production processes are, which have to be closed down or scaled back, among other things, because the supply chain is no longer available as a result of disappearing imports. One of the central questions will be to what extent and how quickly regional production structures can be established and how the economy and society as a whole can become more resilient. In the infrastructure sector, one possibility is the expansion of renewable energies, which are almost always highly decentralised, and the possibility of coupling them with battery systems and thus building up self-sufficient systems at least partially. This must be combined with reinforcement measures in the electricity grid and the integration of intelligent systems (smart grid).

In the context of the Corona crisis, one also becomes acquainted with new behavioral patterns, or appreciates previously known but not yet widely used options. This is especially true for the home office, which suddenly becomes the norm for many. Associated with this is a significant reduction in traffic volume (at national and international level, car traffic as well as air traffic), which at the same time contributes to climate protection and to improving air quality in cities. The latter, incidentally, has a dual significance, since according to statements by the European Public Health Alliance (EPHA) and others, the Corona virus is particularly dangerous for people with pre-existing conditions, and those are often related to poor air quality in cities.

It is to be hoped that, on the one hand, the Corona crisis will be overcome quickly and that as few people as possible will suffer as a result. At the same time, there is a chance that some of the newly learned patterns of behaviour will continue to exist and may have a positive effect in several respects in the future—also in preparation for further pandemics, which cannot be ruled out. With the right orientation of aid measures after the end of the Corona pandemic, the central course can be set for making the state, economy and society more resilient and at the same time taking important steps towards solving the other major transformation challenges, for example through low-carbon investments. Two lessons can be learned from the 2009 economic crisis:

- Firstly, it will clearly be a major challenge for many companies to reinvest and get the economy going after such a significant economic shock. But dealing with the economic and financial crisis at the time has shown that this is not the case: With wise political support, a faltering economic system can be quickly mobilised again. This is why the measures announced by the German Federal Government (and similarly in many other countries) are important and right.
- Secondly, even during the period of the economic and financial crisis, there was a significant reduction in greenhouse gas emissions, but the measures and government incentives implemented thereafter led to such a strong rebound effect that emissions rose even more sharply thereafter. This is something that must be avoided in this case.

In view of the initially limited investment opportunities due to the economic crisis, it is also important to bear in mind that many climate protection measures do not primarily depend on investments, but are rather related to behaviour and lifestyle. Especially with regard to this form of climate protection, many learning effects can be taken away from the crisis.

## Footnotes

1 In the context of the discussion on achieving greenhouse gas neutrality at European level by 2050, the EU Commission clearly emphasises that the energy sector must lead the way in terms of time and must already be virtually greenhouse gas neutral by 2040.
